# The Impact and Cost of Scaling up Midwifery and Obstetrics in 58 Low- and Middle-Income Countries

**DOI:** 10.1371/journal.pone.0098550

**Published:** 2014-06-18

**Authors:** Linda Bartlett, Eva Weissman, Rehana Gubin, Rachel Patton-Molitors, Ingrid K. Friberg

**Affiliations:** 1 Johns Hopkins Bloomberg School of Public Health, Baltimore, Maryland, United States of America; 2 Futures Institute, New York, New York, United States of America; 3 Jhpiego, Washington, DC, United States of America; 4 Maternal Child Health Integrated Program (MCHIP), Baltimore, Maryland, United States of America; Monash University, Australia

## Abstract

**Background and Methods:**

To guide achievement of the Millennium Development Goals, we used the Lives Saved Tool to provide a novel simulation of potential maternal, fetal, and newborn lives and costs saved by scaling up midwifery and obstetrics services, including family planning, in 58 low- and middle-income countries. Typical midwifery and obstetrics interventions were scaled to either 60% of the national population (modest coverage) or 99% (universal coverage).

**Findings:**

Under even a modest scale-up, midwifery services including family planning reduce maternal, fetal, and neonatal deaths by 34%. Increasing midwifery alone or integrated with obstetrics is more cost-effective than scaling up obstetrics alone; when family planning was included, the midwifery model was almost twice as cost-effective as the obstetrics model, at $2,200 versus $4,200 per death averted. The most effective strategy was the most comprehensive: increasing midwives, obstetricians, and family planning could prevent 69% of total deaths under universal scale-up, yielding a cost per death prevented of just $2,100. Within this analysis, the interventions which midwifery and obstetrics are poised to deliver most effectively are different, with midwifery benefits delivered across the continuum of pre-pregnancy, prenatal, labor and delivery, and postpartum-postnatal care, and obstetrics benefits focused mostly on delivery. Including family planning within each scope of practice reduced the number of likely births, and thus deaths, and increased the cost-effectiveness of the entire package (e.g., a 52% reduction in deaths with midwifery and obstetrics increased to 69% when family planning was added; cost decreased from $4,000 to $2,100 per death averted).

**Conclusions:**

This analysis suggests that scaling up midwifery and obstetrics could bring many countries closer to achieving mortality reductions. Midwives alone can achieve remarkable mortality reductions, particularly when they also perform family planning services - the greatest return on investment occurs with the scale-up of midwives and obstetricians together.

## Introduction

Maternal and child mortality levels in many countries are still far from the United Nations Millennium Development Goals (MDGs), yet the MDGs expire in less than two years. Since 1990, maternal mortality has been reduced by 47%, a full quarter short of the three-quarters goal; and under-five mortality, of which an estimated 40% is neonatal deaths, has been reduced by just 35%, about halfway to the two-thirds goal [1]. An inter-agency group assessing progress toward the MDGs determined that 25 of 74 developing countries have made insufficient or no progress in reducing maternal mortality, and that 19 of the 25 countries started with–and continue to have–a maternal mortality ratio greater than 100 deaths per 100,000 live births [2]. Likewise, 51 of the same 74 countries were not on track to achieve the MDG for under-five mortality. These 74 countries also account for approximately 93% of global stillbirths (fetal deaths). Little time remains to accelerate progress toward the MDGs and to inform post-MDG decisions.

The global health community has been advocating for almost a decade for the scale-up of skilled birth attendance in low- and middle-income countries (LMICs) as a way to achieve the MDGs related to child and maternal health. Indeed, skilled birth attendance–the use of competent, professional health providers such as midwives, doctors, and nurses who have been educated and trained in the management of normal pregnancies, childbirth, and the immediate postnatal period and in the identification, management, and referral of complications [3]–is well-recognized for its “triple return” on investment, averting not only maternal and newborn deaths, but fetal deaths as well [2]. Skilled birth attendance is a complex and context-dependent intervention, however, so little experimental evidence exists about its scale-up [3], and skepticism persists about the probability of any expansion in coverage in the near future [4]. To help fill the evidentiary void and increase momentum for scale-up of the two chief components of skilled birth attendance, midwifery and obstetrics [5], we used the latest data and analytical tools available to model the cost and mortality impact that could be achieved in the period from 2012 to 2015 under two different scale-up assumptions.

Specifically, we employed the Lives Saved Tool (*LiST*), which estimates the number of deaths averted by different health interventions, to model different levels of scale-up of midwifery and obstetrics in the 58 countries included in the *State of the World’s Midwifery 2011* (SoWMy) report with either maternal and newborn care (MNC) interventions alone or with MNC interventions plus family planning (FP) [6]–[9]. Linking interventions in *LiST* to midwifery and obstetric competencies and to cost estimates for intervention inputs, we were able to project both the numbers of maternal, fetal, and neonatal lives that would be saved and the total costs of and costs per death averted through a scale-up of midwifery and obstetrics, stratified by coverage scale-up level, cadre, and initial basic emergency obstetric and newborn care (BEmONC) availability.

We were able to link *LiST* interventions with BEmONC and the two additional signal functions of comprehensive emergency obstetric and newborn care (CEmONC)–packages of treatments that, individually, have been proven effective against the key complications of pregnancy and childbirth [10]–with the distinct services performed by midwives and obstetricians, respectively. BEmONC and CEmONC are firmly established in United Nations guidelines and provide a mechanism for change [10]. We expect our findings regarding midwifery and obstetric coverage to inform cost and human resource allocations for BEmONC and CEmONC coverage in the remaining years of the MDGs and to contribute to discussions about post-MDG priorities for maternal, fetal, and neonatal health.

## Methods

Although the focus of our analysis was on scaling up coverage of midwifery and obstetrics, *LiST* does not classify interventions by the cadre of health personnel that can perform them. Therefore, we identified the individual interventions in *LiST* that midwives or obstetricians provide by matching interventions to the competencies identified for midwives by the International Confederation of Midwives (ICM) and for obstetricians by documents from the International Federation of Obstetricians and Gynecologists, and then discussing our findings with an expert opinion group (described below). Midwives and obstetricians both require competencies in obstetric care and in multiple aspects of reproductive health care: pre-pregnancy counseling on nutrition; counseling and provision of FP methods; the prevention and treatment of reproductive tract infections and sexually transmitted infections, including HIV/AIDS; and the entire continuum of pregnancy- and labor-related care, including antenatal visits, skilled attendance at birth and after birth (e.g., essential newborn care), and the management of obstetric and neonatal complications and emergencies. Midwives are able to provide all functions of BEmONC, which includes the following seven services, or “signal functions”: administration of (1) parenteral antibiotics, (2) oxytocics, and (3) anticonvulsants; manual removal of (4) the placenta and (5) retained products; (6) assisted vaginal delivery; and (7) newborn resuscitation with mask [6], [11]. Obstetricians can provide these same seven functions plus two additional ones: (1) surgery, including cesarean section and (2) blood transfusion [8], [14].

### Overview of LiST

*LiST* is an evolving software application that estimates the impact on mortality of selected maternal, neonatal, and child health interventions when scaled under different coverage assumptions, using values from credible sources [9], [12]–[14]. *LiST* permits the simultaneous projection of health impacts for a host of biomedical interventions, calculated using a rigorous demographic and epidemiological framework [12], [14]. The *LiST* module has been built into a free software package, Spectrum, and is linked to three additional modules: DemProj, which projects population by age and sex over time, based on United Nations Population Division data on population levels and trends; FamPlan, which uses FP data from the Demographic and Health Surveys and the proximate determinants of fertility framework to calculate the effects on fertility rates of increasing contraceptive prevalence; and the AIDS Incidence Module, which contains UNAIDS data on country-specific HIV incidence, prevention, and treatment [15]. *LiST* imports default data, which can be modified by the user, including baseline health intervention coverage values, measures of health status, levels of risk factors and population exposures, and cause-of-death data to predict changes in maternal, neonatal, under-five, and stillbirth mortality over time [12]. *LiST* estimates the effectiveness of each intervention based on systematic reviews and meta-analyses combined with data on the quality of the evidence [8], [12], [14]. Once the user selects desired interventions and final coverage levels, *LiST* generates estimates of the number of maternal, fetal (third-trimester stillbirths), neonatal, and under-five deaths that would be averted from the expansion of the selected interventions to the selected coverage levels [12], [14]. Further information about *LiST* can be accessed at http://www.jhsph.edu/departments/international-health/IIP/list/index.html.

### Baseline LiST Values

We chose to model cost and mortality in all 58 countries surveyed for the SoWMy report in *LiST* (Spectrum version 4.23, beta 14). These 58 countries were selected for inclusion in the report based on their collective burden of maternal mortality–91% of global maternal deaths. We used 2012 as the baseline year for the analyses and 2015 as the target year. Standard *LiST* defaults for each coverage indicator were taken from the most recent and relevant nationally representative surveys available at the time, unless otherwise noted. Country-specific estimates of tetanus toxoid (and other routine childhood vaccine) coverage were obtained from the World Health Organization (WHO) and the United Nations Children Fund’s (UNICEF) consensus estimates [16]. We obtained or estimated data from the SoWMy report on the coverage of both types of care, BEmONC and CEmONC, for the 58 LMICs included in the report [11]. The proportion of births occurring in BEmONC and CEmONC facilities was derived from Module 1 of the SoWMY questionnaire and was assumed to correspond to proportions of births attended by practicing midwives and obstetricians, respectively. Because BEmONC is a subset of CEmONC, facilities that offered CEmONC were assumed to also offer BEmONC. The variables in the questionnaire provided information on the percentage of total facilities performing deliveries supported by BEmONC. Since there was no measure of BEmONC availability in the total population, we adjusted BEmONC proportions by rates of institutional delivery from the most recent nationally representative survey. Twenty-eight countries provided sufficient data in the SoWMy questionnaire on BEmONC coverage for their coverage rate to be used directly. For all other countries, the proportion of BEmONC was assumed to be 25% of the default level, to match the average of the 28 countries with data from the questionnaire. All 58 countries were classified into one of four categories based on BEmONC coverage: “very low” (up to 10%); “low” (10%–19%); “intermediate” (20%–39%); and “high” (greater than 40%) (Table 1 and Table S1).

**Table 1:** Countries included in analyses (n = 58), ordered by BEmONC baseline level.

1– Very Low coverage(<6%)		2– Low coverage(6-<20%)		3– Intermediatecoverage (20–<40%)		4– High coverage(≥40%)	
Country	BaselineBEmONCcoverage	Country	BaselineBEmONCcoverage	Country	BaselineBEmONCcoverage	Country	BaselineBEmONCcoverage
Madagascar	0.2	Pakistan	6.0	Morocco	20.5	Indonesia	40.0
Somalia	0.5	Nigeria	6.1	Mauritania	21.1	Gabon	48.2
Ethiopia	0.6	Liberia	6.5	Zimbabwe	22	Uzbekistan	48.7
Chad	0.7	India	6.8	Bolivia	23.4	Gambia	49.4
Bangladesh	0.8	Kenya	7.0	Senegal	24.8	Tajikistan	53.7
Niger	5.3	Burkina Faso	7.1	Mozambique	24.5	Vietnam	62.8
Sudan	0.9	Comoros	7.5	DRC	26.3		
Cambodia	0.9	Cameroon	7.5	Mali	26.7		
Lao PDR	1.0	Rwanda	7.9	Cote d'lvoire	27.0		
Yemen	1.2	Malawi	8.0	Botswana	27.0		
Haiti	1.3	Tanzania	8.2	South Africa	29.5		
Sierra Leone	1.3	Zambia	8.3	Central African Republic	30.5		
Burundi	1.5	Guinea-Bissau	8.9				
Myanmar	1.5	East Timor	9.4				
Nepal	2.0	Bhutan	10.6				
Afghanistan	2.4	Guyana	11.7				
Benin	5.1	Djibouti	12.9				
Guinea	5.4	Togo	14.9				
		Uganda	15.2				
		Nicaragua	16.6				
		Papua New Guinea	17.5				
		Ghana	19.3				

### Selected LiST Interventions

**Figure 1:**
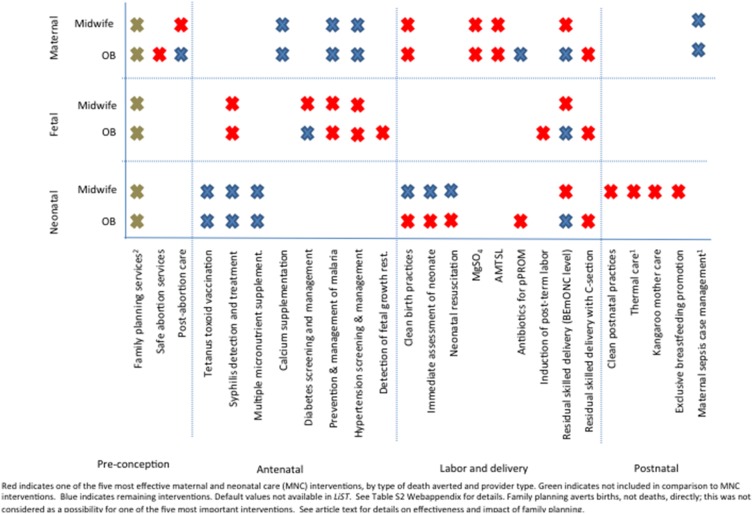
Included *LiST* interventions and type of death averted by provider type.

We selected a total of 26 interventions from *LiST* based on their correspondence to the recognized competencies of midwives, obstetricians, or a combination of the two (Figure 1; additional details on effect sizes and *LiST* interventions in Table S2). We identified the *LiST* interventions most closely aligned with midwifery practice by reviewing the ICM’s list of essential competencies [17] and comparing those activities with the available interventions in *LiST.* All major competencies were represented by *LiST* interventions except for the ICM competency identified as “requisite knowledge and skills.” One competency, maternal sepsis case management, was matched with a *LiST* intervention that had no default effect size; we assumed for this analysis that appropriate application of the intervention would avert 20% of sepsis-related deaths based on the literature [18]. We then identified *LiST* interventions that corresponded to the competencies of obstetricians, some of which were the same as those for midwives, but others of which were not, such as the two non-BEmONC functions of CEmONC (blood transfusion and surgical care, including cesarean section), emergency hysterectomy, surgical contraception (tubal ligation), and second-trimester abortions. Within *LiST*, individual interventions are modeled, with the residual activities of a skilled delivery at the BEmONC level modeled separately (skilled birth attendance and BEmONC signal functions); similarly, for CEmONC, individual interventions are modeled, with the residual activities being modeled as “skilled birth attendance and remaining CEmONC”. The effectiveness of given interventions at the BEmONC and CEmONC levels is not assumed to be the same (Table S2). An expert group consisting of maternal and newborn experts from USAID, the USAID-funded Maternal and Child Health Integrated Program (MCHIP), and Johns Hopkins Bloomberg School of Public Health was convened to consider whether the interventions chosen could actually be delivered in all settings; we could not and did not attempt to account for context-specific variables such as provider competence, access to referral facilities, the location, the time and duration of attendance, or the availability of essential drugs, equipment, and supplies. In other words, we assumed that all cases would receive an appropriate, although not necessarily perfect, standard of care.

### Modeling Scale-up and Lives Saved

We used *LiST* to create 12 unique scenarios as shown in Table 2. Two potential coverage scale-up levels (modest and universal) were modeled. Three potential provider types (midwives, obstetricians, or both) were scaled with one of two scopes of practice: one with only MNC interventions scaled and one with both MNC interventions and family planning. Each scenario generated estimates of maternal, fetal, and neonatal lives saved. The results are presented both in aggregate and by initial BEmONC levels.

**Table 2:** Description of the 12 scale-up scenarios analyzed.

Target coverage level ofinterventions	Cadre of Worker/Skills					
	Midwifery		Obstetrics		Midwifery AND obstetrics	
Modest: 60%	MNC	MNC+FP	MNC	MNC+FP	MNC	MNC+FP
Universal: 99%	MNC	MNC+FP	MNC	MNC+FP	MNC	MNC+FP

MNC: maternal and newborn Care; FP: family planning.

Under the modest scale-up scenarios, each intervention was either scaled up to 60% or, if the baseline coverage level was already greater than 60%, maintained at the current coverage value. On average, this modest increase in providers approximately doubled the coverage of these interventions (Table S1). For the combined midwifery and obstetrics analysis, the higher coverage level of either cadre was used. It was assumed that, in a typical country, midwives would take responsibility for the majority of FP activities, while obstetricians would focus on surgical FP. To model the impact of FP scale-up by midwives, the contraceptive prevalence rate (CPR) was increased either to 20% of the value needed to reach a fertility rate of 2.0 or to 75%, whichever was lower. To approximate the more limited coverage of surgical FP scale-up by obstetricians, an absolute increase of 5% was used regardless of the current CPR, as long as the level of midwifery FP impact was 5% or more; if the level of midwifery FP impact was less than 5%, which often occurred for countries with high CPRs, that lower percentage was applied instead.

Under the universal scale-up scenario, each included intervention was either scaled up to 99% or, if coverage was already at least 99%, maintained at its current value. When scaling up BEmONC interventions for midwives, we assumed that universal coverage would also lead to a small increase (a maximum of 10% of all deliveries) in access to CEmONC. For example, if 89% of deliveries were covered by BEmONC, we assumed that 10% were also covered by CEmONC and that the overall BEmONC coverage was therefore 99%. For FP scale-up by midwives, the CPR was increased either to the value needed to reach a fertility rate of 2.0 or to 75%, whichever was lower. For FP scale-up by obstetricians, the CPR was increased to 20% of the value used for midwives. We chose 2015 as the ending year for each scale-up scenario in order to present relevant results for the MDGs.

### Modeling Cost

Details of the bottom-up, or ingredients-based, costing methodology that has been commonly employed in conjunction with *LiST* have been reported elsewhere [9]. For our analysis, we created a comprehensive list of all costs incurred for the 26 selected interventions that would be required to treat one average person in need. This included all required drugs, supplies, and commodities as well as estimates of the staff time (in minutes) to implement the intervention. Costs for supply inputs were determined using prices from the UNICEF supply catalog and Management Sciences for Health’s international drug price indicator guide [19], [20]. Country-specific labor costs were derived from the WHO-CHOosing Interventions that are Cost Effective (WHO-CHOICE) database [21]. We assigned each case a country-specific clinical overhead cost, obtained from the WHO-CHOICE database, that depended on the length of the clinic visit or, if applicable, hospital stay. We assumed that scale-up would be possible using existing health facilities, so no investments or infrastructure were included. The cost of training additional providers was also not included, even though training would be necessary to achieve the coverage levels modeled.

We estimated the number of women who would require the interventions from a combination of United Nations population data (Spectrum defaults) and incidence data from various sources. The number of women in need of interventions was multiplied by (observed) coverage rates to arrive at an estimated number of women who could be expected to receive a given intervention. The number of likely cases per intervention was multiplied by the average cost for that intervention and aggregated with the other interventions included in the scenario. As with the mortality model, each of the 12 scale-up scenarios of coverage level, provider type, and scope of practice were entered into *LiST* to produce estimates of total scale-up costs and the costs per maternal, fetal, and neonatal death averted. Intervention-specific cost or cost-effectiveness data are not presented, because the impact for each intervention is linked to the coverage of the other interventions in this analysis.

## Results

### Mortality Impact

**Figure 2:**
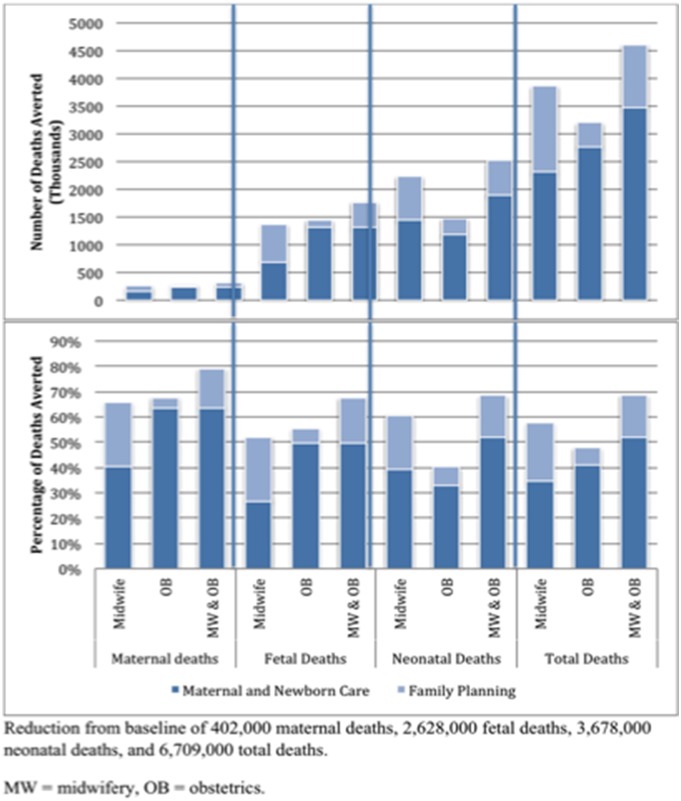
Projected numbers and percentages of deaths averted, by provider and intervention type, under universal coverage.

*LiST* used recent estimates to project that a baseline of approximately 400,000 maternal deaths, 2.6 million fetal deaths, and 3.7 million neonatal deaths–a combined total of nearly 7 million deaths–would occur in the 58 countries included in our model in 2015, assuming a consistent population increase through 2015 and no change in mortality rates or ratios. Deaths averted under all 12 coverage scale-up scenarios can be found in Table 3 and Figure 2. While the scale-up of obstetricians delivering MNC interventions without FP achieved greater total (maternal, fetal, and neonatal) mortality reductions than the scale-up of midwives delivering MNC interventions without FP under both the modest and universal scale-up assumptions (obstetricians: modest = 23%, universal = 41%; midwives: modest = 18%, universal = 34%), midwives achieved greater reductions than obstetricians under either scale-up assumption when FP services appropriate to their capacities were also scaled up (obstetricians: modest = 29%, universal = 48%; midwives: modest = 34%, universal = 58%). Although maternal and fetal death reductions show the same pattern, midwifery achieved substantially greater reductions than obstetrics in neonatal mortality, regardless of the scale-up of FP. For all scenarios, when family planning was added, 1.5 times more lives were saved than in scenarios with MNC alone. However, the greatest impact occurred when midwives and obstetricians worked together and when FP was scaled: 52% of maternal, 43% of fetal, and 44% of neonatal deaths, or 44% of total deaths, could be prevented under the modest scale-up assumption, and a full 79% of maternal, 68% of fetal, and 68% of neonatal deaths, or 69% of total deaths, could be prevented under the universal scale-up assumption. Universal coverage saved an average of 1.7 times more lives than the same scenarios under modest scale-up.

**Table 3:** Projected lives saved and percent reduction by 2015, by coverage scale-up level, provider type, and scope of practice.

		Maternal deaths averted		Fetal deaths averted		Neonatal deaths averted		Total deaths averted	
Coverage scale-Up level		Modest	Universal	Modest	Universal	Modest	Universal	Modest	Universal
**Provider type** **and scope** **of practice**	**Midwife MNC** **only**	94,000^1^(23%)	162,000(40%)	330,000(13%)	700,000(27%)	754,000(21%)	1,449,000(39%)	1,178,000(18%)	2,311,000(34%)
	**OB MNC Only**	165,000(41%)	255,000(63%)	723,000(28%)	1,308,000(50%)	642,000(17%)	1,200,000(33%)	1,530,000(23%)	2,764,000(41%)
	**Midwife MNC + FP**	156,000(39%)	264,000(66%)	790,000(30%)	1,365,000(52%)	1,347,000(37%)	2,235,000(61%)	2,293,000(34%)	3,864,000(58%)
	**OB MNC + FP**	181,000(45%)	272,000(68%)	880,000(33%)	1,452,000(55%)	894,000(24%)	1,476,000(40%)	1,955,000(29%)	3,201,000(48%)
	**Midwife & OB** **MNC Only**	164,000(41%)	255,000(63%)	770,000(29%)	1,308,000(50%)	1,107,000(30%)	1,903,000(52%)	2,041,000(30%)	3,466,000(52%)
	**Midwife & OB** **MNC + FP**	211,000(52%)	318,000(79%)	1,142,000(43%)	1,774,000(68%)	1,626,000(44%)	2,517,000(68%)	2,979,000(44%)	4,609,000(69%)

1 N(% reduction); From a baseline assumed 402,000 maternal deaths, 2.6 million fetal deaths, 3.7 million neonatal deaths and 6.7 million total deaths in 2015. All numbers rounded before calculations. OB: obstetrics; MNC: maternal and neonatal care; FP: family planning.

The level of mortality impact that could be achieved varied greatly depending on the baseline level of BEmONC coverage and was generally greater for countries that started in a lower category of baseline BEmONC coverage (Table 4). Among countries with “very low” baseline BEmONC availability, the proportion of deaths averted ranged from 38% under the modest scale-up assumption to 64% under the universal scale-up assumption. For those with “low” baseline BEmONC coverage, the mortality impact ranged from 34% to 56%, depending on the scale-up assumption. “Intermediate” baseline BEmONC availability yielded between 34% and 65% of deaths averted, and “high” baseline BEmONC availability yielded between 23% and 43%. These reductions in mortality decrease in impact by almost one-third for MNC-only models that exclude FP (Table S3). If universal scale-up of midwifery and FP could be accomplished, 3.9 million of the 6.7 million deaths calculated at baseline could be averted.

**Table 4:** Total deaths averted by midwifery scale-up, by country BEmONC coverage classification, including family planning.

	Baseline coverage of midwifery interventions, including BEmONC				
	Very low	Low	Intermediate	High	All
**Baseline deaths**	1,439,000	4,295,000	624,000	350,000	6,709,000
**Deaths averted with modest (60%) scale-up of** **midwifery**	540,000 (38%)^1^	1,462,000 (34%)	210,000 (34%)	81,000 (23%)	2,293,000 (34%)
**Deaths averted with universal (99%) scale-up of** **midwifery**	923,000 (64%)	2,385,000 (56%)	404,000 (65%)	151,000 (43%)	3,864,000 (58%)

1 Percent reduction from no-change scenario.

Impact on mortality varied by region as well (Table 5). Greater impact on mortality is seen in Africa than in Asia. At modest scale up, 8% more deaths would be averted in Africa; and with universal coverage, almost a quarter more deaths may be prevented compared to Asia (39% vs. 31%; 71% vs. 48%).

**Table 5:** Total deaths averted by midwifery scale-up, including family planning, by region.

	Baseline coverage of midwifery interventions, including BEmONC			
	Africa	Asia	Other	All
**Baseline deaths**	2,747,000	3,927,000	34,000	6,709,000
**Deaths averted with modest (60%) scale-up of** **midwifery**	1,059,000 (39%)	1,220,000 (31%)	13,000 (38%)	2,293,000 (34%)
**Deaths averted with universal (99%) scale-up** **of midwifery**	1,962,000 (71%)	1,884,000 (48%)	19,000 (56%)	3,864,000 (58%)

We also identified the top five interventions, in addition to FP, that had the greatest impact on each mortality type, when stratified by provider type (Figure 1). Each of the interventions is a standalone action, and each intervention performed during labor and delivery had a separate effect size (Table S2). For maternal deaths, most of the critical interventions were common to both provider types; that is, either provider type could perform them and achieve a high impact, although there was additional impact with CEmONC provided by only obstetricians. The interventions with the greatest impact on maternal deaths were concentrated in safe abortion services and postabortion care, and in labor and delivery. For fetal and neonatal deaths, midwives and obstetricians had fewer similarities in the interventions with the greatest impact - the top interventions by obstetricians were concentrated in labor and delivery, while those for midwives were concentrated in antenatal and postnatal care (for neonates only).

### Cost Impact

**Figure 3:**
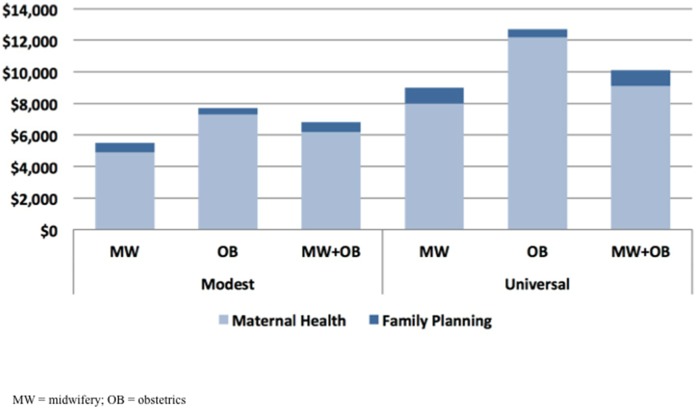
Total cost of scale-up by provider type.

We examined the additional costs that would be incurred by 2015 for the modest and universal scale-up of each scenario. Results are shown in Figure 3. Providing services exclusively through midwives was the least costly option under both the modest and universal scale-up assumptions (an additional $5.5 and $9 billion, respectively), and providing services exclusively through obstetricians was the most costly option (an additional $7.7 and $12.7 billion). When midwives and obstetricians worked together, costs were substantially lower than for obstetricians alone ($6.8 compared to $10 billion), regardless of the level of coverage scale-up.

**Figure 4:**
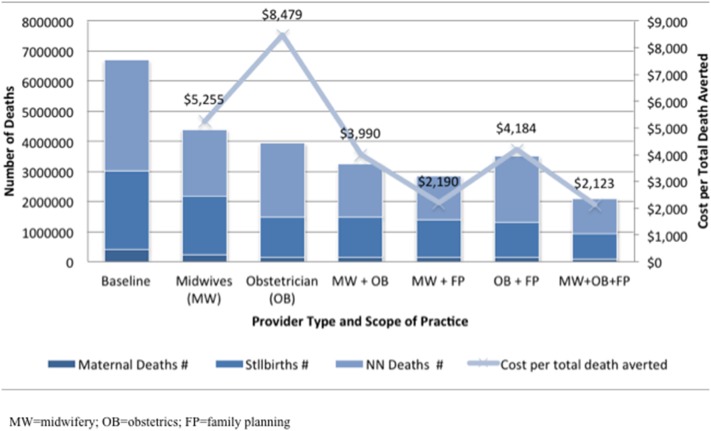
Projected numbers of deaths averted under universal coverage, with costs per total deaths averted.

The cost per death averted is shown in Figure 4 and Table S3, along with the estimated number of each type of death remaining, under universal (99%) coverage scenarios. Although the cost per death averted is greater for the obstetricians alone, obstetricians also avert more deaths than midwives alone, when only MNC interventions are considered. The combined utilization of both midwives and obstetricians was both the most cost-effective and the most effective in reducing deaths. When including family planning, the picture is more multifaceted. The cost per maternal death averted by scaling up midwifery and FP was $32,100, compared with $49,200 by scaling up obstetricians and FP. When maternal deaths, which were the most costly to avert, were combined with fetal and neonatal deaths, the midwifery scale-up model with FP was almost twice as cost-effective as the obstetrics model, at $2,200 versus $4,200 per death averted. However, in the model that scaled both midwives and obstetricians and also scaled FP, the cost per death averted was the lowest, at just $2,100.

In all coverage scale-up and provider type scenarios, large cost reductions could be achieved when MNC interventions were accompanied by an FP scale-up. In the midwifery universal scale-up scenario, for instance, total costs decreased by $1.4 billion (reduced from $15.5 to $14.1 billion) when FP was scaled. In the combined midwifery and obstetrics universal scale-up scenario, the cost savings were more than $4 billion (reduced from $19.3 to $15.2 billion). On average, when the scenarios excluded FP, the cost per death averted was more than twice that in those with FP added.

## Discussion

With only a modest scale-up of midwifery alone, and with no attempt to scale family planning, *LiST* predicted that nearly 1.2 million maternal, fetal, and neonatal lives could be saved by 2015 in countries struggling to reach the maternal and child health MDGs, a mortality reduction of 18% [22]. The numbers become more astounding as the scale-up scenarios reach universal coverage, add the scale-up of FP, and add the scale-up of obstetricians in addition to midwives alone. In the best case, with all of those elements included, an estimated 4.6 million lives could be saved, or a 79% reduction in maternal mortality and 68% reduction in fetal and neonatal mortality, all of which are beyond the reductions sought by the MDGs. The impact was higher in the lowest resource settings: countries with the lowest baseline BEmONC; and in Africa where there is less coverage of life saving interventions at baseline: obstetric care and lesser contraceptive prevalence, thus greater potential for improvement [23].

The costs of these scale-up scenarios are large, but *LiST* demonstrated that universal coverage of midwifery, obstetrics, and family planning resulted in a cost per death averted for maternal, fetal, and neonatal deaths combined of a mere $2,100.

Our findings show that the scale-up of midwifery could prevent more neonatal deaths than the scale-up of obstetrics alone, while obstetricians can prevent a greater number of maternal and fetal deaths. These results suggest that midwifery alone can be an efficient *and* cost-effective option for achieving large mortality reductions. Midwives are also able to perform a host of other services during the continuum of care from household to hospital, which makes them as valuable to women and children as obstetricians. One of these midwifery competencies, breastfeeding counseling, is the main reason that midwives alone achieved greater reductions of neonatal deaths than obstetricians alone [9], [24]. Similarly, it is the ability to perform a cesarean section that results in obstetricians preventing more maternal and fetal deaths [9].

The most effective provider type, however, was neither midwives nor obstetricians alone, but the two cadres working together. We assume that the combination was more effective because the two providers would task-shift to carry out only those competencies for which each provider type was more capable, efficient, and successful. This evidence supports the contention of Graham et al. that there is an optimal “partnership ratio”–that is, a mix between midwives and medical professionals that can achieve the greatest mortality reductions because optimal proportions of skilled attendants for the number of normal, complicated, and emergency cases is more important than the absolute number or even the competence of those attendants [5]. Our findings of the most consistently effective interventions contribute additional information about the ideal care environment and suggest that there are certain interventions that should be emphasized for each provider type. The combination of midwives and obstetricians together also costs considerably less than using obstetricians alone, likely because of the more cost-effective distribution of the workload.

Family planning is also a critical intervention for reducing pregnancies and births, and therefore deaths. If family planning utilization is increased, scaling up midwives theoretically could prevent just as many maternal and fetal deaths as the scale-up of obstetricians without family planning, since FP is one of the core competencies of midwifery, while less central to the activities of obstetricians. When FP was included in any scenario, the cost-effectiveness of the scenario doubled because of the attendant decrease in the number of deaths combined with the reduction in maternal care costs due to reduced deliveries. Although our cost methodology was limited by, among other things, the types of inputs that we included and the available cost data on those inputs, our relative cost-per-death-averted estimates are affected equally by those limitations and therefore still produce instructive comparisons.

There are a number of additional economic arguments for using midwives instead of obstetricians to provide BEmONC-level maternal and newborn care and FP. Although not part of this modeling exercise, the cost of training midwives is substantially lower than that of training obstetricians, and pre-service training for midwives can be completed in an average of three years, versus at least six years for obstetricians. According to WHO, pre-service training costs for a midwife are about one-third of a physician’s (i.e., an average of 17.6 times a country’s gross domestic product per capita for a midwife versus an average of 36.9 times for a physician) [25], [26]. In addition, in many countries, attrition among midwives has been found to be half that of physicians [27].

The main limitation of our analysis is our reliance on a modeling tool: *LiST* projects data where none would otherwise exist, so its findings should not be given the same weight as observational or experimental evidence. The default indicators contained in *LiST* vary in the quality and quantity of their underlying evidence [12], and because *LiST* does not contain cadre-specific coverage indicators, we chose to substitute data on BEmONC and CEmONC coverage from the SoWMy report, so our findings are limited by that data as well. Only 30 of the 58 countries in the SoWMy report provided full BEmONC coverage data, so the remainder of the data used were based on approximations. The substitution of BEmONC and CEmONC data for midwifery and obstetric coverage is itself a limitation, but because midwives should have the competencies necessary to provide full-service BEmONC, in addition to the other competencies identified in Figure 1, we considered BEmONC an appropriate proxy for midwifery and CEmONC an appropriate proxy for specialists. Certain CEmONC services could also arguably be provided by general physicians, not merely by specialist obstetricians. In addition, *LiST* impact estimates are based on systematic literature reviews and extensive expert opinion exercises, thus arguably using the best estimates available for intervention impact when no empiric data exists. Finally, *LiST* is not yet capable of providing uncertainty estimates for its projections, so we were unable to show the level of confidence that we had in any given outcome.

In addition to its technical restrictions, *LiST* contains two assumptions that should lead to cautious application of its projections. First, *LiST* assumes not only that the chosen interventions are present in a given environment but that they can and will be utilized in the proportions necessary to achieve a given coverage level [7]. In fact, coverage depends heavily on provider quality and competence and on equitable access to inputs and referrals; for example, the best-trained providers with the most resources and options for referral are more likely to be concentrated in urban areas than in rural ones, and in areas of greater wealth than less. Furthermore, while *LiST* has been used to evaluate strategies that are specifically targeted at more vulnerable groups, and therefore attempts to address issues of equity [28], [29], our model did not account for inequities and instead assumed the equitable distribution of and equitable access to all interventions. Second, *LiST* assumes that all subnational groups have similar levels and causes of mortality [7]. These both might result in incorrect projections, since groups with different baseline mortality levels or differential access to BEmONC might not achieve similar overall reductions. Another important consideration is that reaching the people in the most remote or difficult to reach areas would be more challenging programmatically and also more expensive. However, assuming that greater mortality risk occurs in areas where there is less access to obstetric care, the extra resources to reach them would also very likely achieve a proportionally greater impact on deaths averted.

Although there have been isolated experimental studies regarding the scale-up of midwifery and obstetrics, a model such as *LiST* offers the opportunity to estimate potential impacts on a global scale and to generate meaningful data for discussion of international health goals and how to achieve them. Our choice of scale-up period was necessarily short because of the impending MDG deadlines, and our universal scale-up assumption is clearly aspirational, but our findings nevertheless illustrate that a decision to scale up midwifery and obstetrics in LMICs will almost certainly result in large numbers of maternal, fetal, and neonatal lives saved, at a lower cost than might previously have been assumed.

The *LiST* model demonstrates that scaling up midwifery and obstetrics could bring many LMICs closer to achieving the maternal, fetal, and neonatal mortality reductions outlined in MDG 4 and 5. Scaling up midwives who are also able to provide FP and who work within the health system and with obstetricians is the most cost-effective way to prevent deaths. However, midwives alone can achieve remarkable mortality reductions, particularly if they perform FP services, at a substantially lower cost than obstetricians. The scale-up of family planning is critical to reaching the greatest mortality reductions, but there are a number of other high-impact interventions that should be emphasized in any scale-up program. As the deadline for achieving the MDGs approaches and new goals for maternal and child health are set, the interwoven scale-up of midwifery and obstetrics must continue to be a large part of the conversation.

## Supporting Information

Table S1 **Excel data file.**
(XLSX)

Table S2 **Effect sizes and List documentation.**
(PDF)

Table S3 **Additional results.**
(PDF)
