# Supplementary material for: The Impact and Cost of Scaling up Midwifery and Obstetrics in 58 Low- and Middle-Income Countries
Source: PLoS One. 2014 Jun 18;9(6):e98550. doi: 10.1371/journal.pone.0098550 (PMC4062394; doi:10.1371/journal.pone.0098550)
Supplement: Table S2 — Effect sizes and List documentation. (PDF) [file pone.0098550.s002.pdf]

Webappendix B: Effect Sizes and LiST documentation

The Lives Saved Tool (*LiST*) is a multi-cause model of mortality. It uses the most current estimates of mortality (both causes and levels) along with coverage data on health interventions, and links these with predicted future coverage and estimates of how effective these health interventions are in reducing cause specific mortality over time. *LiST* is part of the Spectrum Policy Modeling Software. The Spectrum policy modeling software includes multiple modules in addition to *LiST*. These include DemProj (a demography module), AIM (the AIDS Impact Module) and FamPlan (a family planning module). Each of these link to the LiST module to produce estimate of deaths and deaths averted. The Family Planning module uses the proximate determinants model to estimate how mortality will change as a result of increasing family planning coverage, among other potential indicators. For additional details on how family planning affects the total fertility rate, see the Family Planning Manual (www.futuresinstitute.org/spectrum2.aspx#famplan; futuresinstitute.org/Download/Spectrum/Manuals/FampmanE.pdf)

Delivery care is separated into several different interventions, with individual interventions having proven impact on mortality separated out whenever possible. The residual activities which are required at the BEmONC or CEmONC level are grouped together under the title ‘skilled birth attendance’. The effect size for an individual intervention may be identical if it is assumed that the intervention can be delivered equally as well at multiple different locations and with multiple different providers. Alternatively, they may be different if it is clear that there is a different impact based on provider/location.

Another important point about the modeling relates to delivery care interventions. It is assumed that although coverage of a skilled attendant could be increased to a given level, intermediate levels of coverage do not insure that this attendant always uses the interventions accurately and appropriately. These standard assumptions about quality of care are built into the *LiST* model and displayed at the end of this document.

Below are listed all possible effectiveness estimates within the *LiST* portion of the software. Those in red are included in the current analysis, while those in blue indicate that a non-standard effect size was used. Explanations are in the footnotes of each table. And finally, the black are interventions which were not modeled in the current analysis.

Maternal Effect Sizes

Cause of Death	Intervention	Effect

Cause of Death	Intervention	Effec t

Antepartum Hemorrhage	Skilled birth attendance	BEmONC level – No C-section	.2
		CEmONC level With C-section	.8
Postpartum Hemorrhage	Active management of the third stage of labor{X}		.27
	Skilled birth attendance	BEmONC level – No C-section	.65
		CEmONC level With C-section	.95
Hypertensive diseases	Calcium supplementation[2]		.23
	Magnesium sulfate for pre-eclampsia[2][1]		.59
	Magnesium sulfate for eclampsia[2]		.41
	Labor and Delivery Care – CEmONC level[2]		.96
	Hypertensive disease case management [1]		NA
Sepsis Infections	Antibiotics for pPRoM		.26 (.1)
	Clean birth practices	home, SBA	.1 (.5)
		facility, SBA	.1 (.5)
		BEmONC level – No C-section	.5
		CEmONC level With C-section	.7
	Sepsis case management [2]		NA
Note: All affected fractions are equal to 1 unless otherwise stated. All numbers in parentheses are the relevant affected fractions<br>NA: This is a placeholder for a future intervention.			

Abortion	Safe abortion services		.95
	Post abortion case management		.90
Obstructed labor	Skilled birth attendance	BEmONC level – No C-section	.08
		CEmONC level With C-section	.99
Ectopic pregnancy	Ectopic pregnancy case management		.3
Malaria	IPTp		.4
	Case management of malaria		.84
Other indirect causes	Tetanus toxoid immunization		.98 (.005)

[1]Hypertensive disease case management is a place holder for management of moderate hypertensive disease and we have no valid effect size for this at the time the time of the analysis. ‘MgSO4 for pre-eclampsia’ in reality refers to management of hypertensive disease including the availability of MgSO4 if needed. Thus we modeled the second indicator only.
[2]Sepsis case management had no available effect size at the time of the analysis. It was decided to be extremely conservative in the estimate of this potential effect, since it was also decided that it was necessary to include this effect for a comprehensive analysis. The value used was 0.25.

Stillbirth Effect Sizes

Cause of Death	Intervention	Effect
Antepartum Stillbirths	Periconceptual folic acid supplementation or fortification	.41
	IPT malaria during pregnancy (IPTp)[4]	.22
	Syphilis detection and treatment[5]	.82
	Detection and treatment of hypertensive diseases in pregnancy[1]	.20
	Detection and treatment of diabetes of pregnancy[6]	.10
	Detection and management of fetal growth restriction[7]	.20
	Identification and induction of mothers with >= 41 weeks gestation[8]	.69 (.036)

Note: All affected fractions are equal to 1 unless otherwise stated. All numbers in parentheses are the relevant affected fractions

[1]This was actually modeled as MgSO4 management of pre-eclampsia although the effect size is the effect of detection and treatment of hypertensive diseases in pregnancy.

Cause of Death	Intervention		Effect
Intrapartum Stillbirths	Periconceptual folic acid supplementation or fortification[3]		.41
	Detection and treatment of hypertensive diseases in pregnancy		.20
	Detection and treatment of diabetes of pregnancy[6]		.10
	Detection and management of fetal growth restriction[7]		.20
	Identification and induction of mothers with >= 41 weeks gestation[8]		.69 (.036)
	Skilled birth attendance[9]	home, SBA	.23
		facility, SBA	.23
		BEmONC level – No C-section	.45
		CEmONC level With C-section	.75

Neonatal Effect Sizes

Cause of Death	Intervention		Effect
Diarrhea	ORS[11]		0.93
Sepsis	Syphilis detection and treatment[5]		0.025
	Antibiotics for pPRoM[13]		0.08
	Clean birth practices[15]	home, no skilled attendant	.15
		home, SBA	.23
		facility, SBA	.27
		BEmONC level – No C-section	.27
		CEmONC level With C-section	.27
	Clean postnatal practices[15]		0.31
	Case management of severe infection[17]	Oral antibiotics	0.42
		Injectible antibiotics	0.68
		Full supportive care	0.83
Pneumonia	Case management of severe infection[17]	Oral antibiotics	0.42
		Injectible antibiotics	0.68
		Full supportive care	0.83
Asphyxia	Immediate assessment and stimulation[14]		0.1
	Skilled birth attendance[16]	home, SBA	0.25
		facility, SBA	0.25
		BEmONC level – No	0.40
		CEmONC level With C-section	0.85
	Neonatal	home, SBA	0.2

Cause of Death	Intervention		Effect
Prematurity	Antenatal corticosteroids for preterm labor[12]		0.53
	Antibiotics for pPRoM[13]		0.12
	Immediate assessment and stimulation[14]		0.1
	Skilled birth attendance[16]	home, SBA	0.1
		facility, SBA	0.1
		BEmONC level – No C-section	0.1
		CEmONC level With C-section	0.1
	Neonatal resuscitation[14]	home, SBA	.05
		facility, SBA	.1
		BEmONC level – No C-section	.1
		CEmONC level With C-section	.1
	Thermal care[1]		NA
	Kangaroo mother care[18]		0.51
	Case management of severe neonatal infection - full supportive care[17]		0.28
Tetanus	Tetanus toxoid[10]		0.94
	Clean birth practices[15]	home, no skilled attendant	0.3
		home, SBA	.35
		facility, SBA	.38
		BEmONC level – No	0.38
		CEmONC level With C-section	0.38
Congenita	Periconceptual Folic Acid[19]		0.35

	resuscitation [14]	facility, SBA	0.3
		BEmONC level – No C-section	0.3
		CEmONC level With C-section	0.3
	Case management of severe neonatal infection - full supportive care[17]		0.05

l anomalies		
Other	Case management of severe neonatal infection - full supportive care[17]	0.10
Note: All affected fractions are equal to 1. NA: This is a place holder for a future intervention.		

[1]Thermal care effect size was estimated in reference X, but was deemed inconclusive, thus not included in the standard model. However, this effect is likely to be true, so it was included in this analysis. The value used was 0.20. The reference is (X)

Additional Neonatal Effects

Risk Factor	Intervention	Effect
On IUGR	IPT malaria during pregnancy (IPTp)[20]	0.35
	Balanced energy supplementation[21]	0.32
	Multiple micronutrient supplementation[22]	0.09

The affected fraction for IPTp is the proportion of 1st and 2nd pregnancies exposed to malaria. The affected fraction for balanced energy supplementation is the proportion of the population living under the poverty line, or $1.25 per day. The affected fraction for multiple micronutrient supplementation is 1.

Cause of Death	Risk Factor	Odds Ratio
Diarrhea	IUGR/Low birth weight [23]	2
	Not IUGR/Low birth weight	1
Sepsis/Pneumonia	IUGR/Low birth weight[23]	2
	Not IUGR/Low birth weight	1
Asphyxia	IUGR/Low birth weight[23]	2.3
	Not IUGR/Low birth weight	1
Diarrhea	Exclusive breastfeeding[24]	1
	Partial breastfeeding	2.28
	Predominant breastfeeding	4.62
	Not breastfeeding	10.53
Sepsis/Pneumonia	Exclusive breastfeeding[23]	1
	Partial breastfeeding	1.75
	Predominant breastfeeding	2.49
	Not breastfeeding	15.13

1 refers to the reference population.

Risk Factor	Risk Factor/ Intervention	Odds

		Ratio
On stunting	IUGR/Low birth weight	21.6
	Not IUGR/Low birth weight	1
	Diarrhea (per episode)[25]	1.04
	No Diarrhea	1
On appropriate breastfeedin g	Breastfeeding promotion	4
	No promotion	1

1 refers to the reference population.

Delivery Related Associations

	**Coverage Values**		Delivery Values as seen in *LiST*				
**Country Range**	**Skilled Attendan t**	**Instituti onal delivery**	No Assistanc e	SBA in home	Essential care for all women and immediate care at birth	BEmOC	CEmOC
0-29% institutional delivery			100-Inst	SBA-Ins t	90%*Inst Del	0%*Inst	10%*Inst
eg	10.00	9.00	90.00	1.00	8.10	0.00	0.90
30-49% institutional delivery			100-Inst	SBA-Ins t	50% Inst Del	30%*Inst	20%*Inst
eg	38.00	35.00	62.00	3.00	17.50	10.50	7.00
50-94% institutional delivery			100-Inst	SBA-Ins t	25%*Inst	15%*Inst	60%*Inst
eg	90.00	75.00	10.00	15.00	18.75	11.25	45.00
+95% institutional delivery			100-Inst	SBA-Ins t	0%*Inst	0%*Inst	100%*Inst
eg	100.00	95.00	0.00	5.00	0.00	0.00	95.00

	No Assistanc e	SBA in home	Essential care for all women and immediate care at birth	BEmOC	CEmOC
Clean Delivery Practices	0*No	50*SBA	60*Essential	85*BEMO C	95*CEMOC

Immediate assessment and stimulation	0*No	25*SBA	50*Essential	80*BEMOC	90*CEMOC
Labor and Delivery Management	0*No	100*SBA	100*Essential	100*BEMOC	100*CEMOC
Neonatal resusctiation	0*No	0*SBA	0*Essential	20*BEMOC	70*CEMOC
Steroids for preterm labour	0*No	0*SBA	20*Essential	85*BEMOC	95*CEMOC
Antibiotics for pPROM	0*No	0*SBA	20*Essential	85*BEMOC	95*CEMOC
MgSO4 for eclampsia	0*No	0*SBA	20*Essential	85*BEMOC	95*CEMOC
AMTSL	0*No	0*SBA	20*Essential	85*BEMOC	95*CEMOC
Induction of labor for post-term	0*No	0*SBA	0*Essential	0*BEMOC	20*CEMOC

Inst=Institutional Delivery
SBA=Skilled birth attendant
BEmONC=Basic Emergency Obstetric Care
CEmONC=Comprehensive Emergency Obstetric Care

Reference List

[1] Lawn JE, Wilczynska-Ketende K, Cousens S. Estimating the causes of 4 million neonatal deaths in the year 2000. Int J Epidemiol 2006 Mar 23;35:706-18.

[2] Ronsmans C, Campbell O. Quantifying the fall in mortality associated with interventions related to hypertensive diseases of pregnancy. BMC Public Health 2011 Apr 13;11(Suppl 3):S8.

[3] Imdad A, Yakoob MY, Bhutta ZA. The effect of folic acid, protein energy and multiple micronutrient supplements in pregnancy on stillbirths. BMC Public Health 2011 Apr 13;11(Suppl 3):S4.

[4] Ishaque S, Yakoob MY, Imdad A, Goldenberg RL, Eisele TP, Bhutta ZA. Effectiveness of interventions to screen and manage infections during pregnancy on reducing stillbirths: a review. BMC Public Health 2011 Apr 13;11(Suppl 3):S3.

[5] Blencowe H, Cousens S, Kamb N, Berman S, Lawn JE. Detection and treatment of syphilis in pregnancy to reduce syphilis related stillbirths and neonatal mortality. BMC Public Health 2011 Apr 13;11(Suppl 3):S9.

[6] Syed M, Javed H, Yakoob MY, Bhutta ZA. Effect of screening and management of diabetes during pregnancy on stillbirths. BMC Public Health 2011 Apr 13;11(Suppl 3):S2.

[7] Imdad A, Yakoob MY, Siddiqui S, Bhutta ZA. Screening and triage of intrauterine growth restriction (IUGR) in general population and high risk pregnancies: a systematic review with a focus on reduction of IUGR related stillbirths. BMC Public Health 2011 Apr 13;11(Suppl 3):S1.

[8] Hussain AA, Yakoob MY, Imdad A, Bhutta ZA. Elective induction for pregnancies at or beyond 41 weeks of gestation and its impact on stillbirths: a systematic review with meta-analysis. BMC Public Health 2011 Apr 13;11(Suppl 3):S5.

[9] Yakoob MY, Ali MA, Imdad A, Lawn JE, van den Broek N, Bhutta ZA. The effect of providing skilled birth attendance and emergency obstetric care in preventing stillbirths. BMC Public Health 2011 Apr 13;11(Suppl 3):S7.

[10] Blencowe H, Lawn JE, Vandelaer J, Roper M, Cousens S. Tetanus Toxoid immunisation to reduce mortality from neonatal tetanus. Int J Epidemiol 2010;39:i102-i109.

[11] Munos M, Fischer-Walker C, Black RE. The Effect of Oral Rehydration Solution and Recommended Home Fluids on Diarrhea Mortality. Int J Epidemiol 2010;39:i75-i87.

[12] Mwansa-Kambafwile J, Cousens S, Hansen T, Lawn JE. Antenatal steroids in preterm labour for the prevention of neonatal deaths due to complications of preterm birth. Int J Epidemiol 2010;39:i122-i133.

[13] Cousens S, Blencowe H, Gravett M, Lawn JE. Antibiotics for preterm prelabour rupture of membranes: prevention of neonatal deaths due to complications of preterm birth and infection. Int J Epidemiol 2010;39:i134-i143.

[14] Lee ACC, Cousens S, Wall S, et al. Neonatal resuscitation and immediate newborn assessment and stimulation for the prevention of neonatal deaths: a systematic review and Delphi estimation of mortality effect. BMC Public Health 2011 Apr 13;11(Suppl 3):S12.

[15] Blencowe H, Cousens S, Mullany LC, et al. Clean birth and postnatal care practices to reduce neonatal deaths from sepsis and tetanus. Not published.

[16] Lee ACC, Cousens S, Darmstadt GL, et al. Care during labor and birth for the prevention of intrapartum-related neonatal deaths: a systematic review and Delphi estimation of mortality effect. BMC Public Health 2011 Apr 13;11(Suppl 3):S10.

[17] Zaidi AKM, Ganatra HA, Syed S, et al. Effect of case management on mortality from neonatal sepsis and pneumonia. BMC Public Health 2011 Apr 13;11(Suppl 3):S13.

[18] Lawn JE, Mwansa-Kambafwile J, Horta BL, Barros FC, Cousens S. "Kangaroo mother care" to prevent neonatal deaths due to preterm birth complications. Int J Epidemiol 2010;39:i144-i154.

[19] Blencowe H, Cousens S, Modell B, Lawn JE. Folic Acid to reduce neonatal mortality from neural tube disorders. Int J Epidemiol 2010;39:i110-i121.

[20] Eisele TP, Larsen D, Steketee RW. Protective efficacy of interventions for preventing malaria mortality in children in Plasmodium falciparum endemic areas. Int J Epidemiol 2010;39:i88-i101.

[21] Imdad A, Bhutta ZA. Effect of balanced protein energy supplementation during pregnancy on birth outcomes. BMC Public Health 2011 Apr 13;11(Suppl 3):S17.

[22] Haider BA, Yakoob MY, Bhutta ZA. Effect of multiple micronutrient supplementation during pregnancy on maternal and birth outcomes. BMC Public Health 2011 Apr 13;11(Suppl 3):S19.

[23] Black RE, Allen LH, Bhutta ZA, et al. Maternal and child undernutrition: global and regional exposures and health consequences. Lancet 2008 Jan 19;371(9608):243-60.

[24] Lamberti LM, Fischer-Walker C, Noiman A, Victora C, Black RE. Breastfeeding and the risk for diarrhea morbidity and mortality. BMC PUblic Health 2011 Apr 13;11(Suppl 3):S15.

[25] Checkley W, Buckley G, Gilman RH, et al. Multi-country analysis of the effects of diarrhoea on childhood stunting. Int J Epidemiol 2008 Aug;37(4):816-30.
