# Supplementary material for: The Impact and Cost of Scaling up Midwifery and Obstetrics in 58 Low- and Middle-Income Countries
Source: PLoS One. 2014 Jun 18;9(6):e98550. doi: 10.1371/journal.pone.0098550 (PMC4062394; doi:10.1371/journal.pone.0098550)
Supplement: Table S3 — Additional results. (PDF) [file pone.0098550.s003.pdf]

WebAppendix C.
Additional Results

WebTable 1. Total deaths averted by midwifery scale-up by country BEmONC coverage classification excluding family planning.

	Baseline Coverage of Midwifery Interventions, including BEmONC				
	**Very Low**	**Low**	**Intermediate**	**High**	**All**
**Baseline Deaths**	1,439,000	4,295,000	624,000	350,000	6,709,000
**Deaths Averted with Modest (60%) Scale-up of Midwifery**	300,000 (21%)[1]	728,000 (17%)	108,000 (17%)	42,000 (12%)	1,178,000 (18%)
**Deaths Averted with Universal (99%) Scale-up of Midwifery**	546,000 (38%)	1,455,000 (34%)	213,000 (34%)	97,000 (28%)	2,311,000 (34%)

[1]Percent reduction from no change scenario.

Web Table 2. Costs per death averted by mortality type, provider type, and scope of practice.

		Maternal Deaths		Stillbirths		Neonatal Deaths		Total Deaths	
**Scenario**		**Deaths Averted**	**Cost/death Averted**	**Deaths Averted**	**Cost/death Averted**	**Deaths Averted**	**Cost/death Averted**	**Deaths Averted**	**Cost/death Averted**
**Excluding Family Planning**	**Midwives**	161,500	$75,200	700,300	$20,400	1,449,100	$8,400	2,311,000	$5,300
	**Obstetricians**	255,400	$91,800	1,308,300	$17,900	1,200,200	$19,500	2,763,900	$8,500
	**Midwives + Obstetricians**	255,400	$54,100	1,308,100	$10,600	1,902,700	$7,300	3,466,200	$4,000
**Including Family Planning**	**Midwives**	263,800	$32,100	1,364,900	$6,200	2,235,300	$3,800	3,864,100	$2,200
	**Obstetricians**	272,200	$49,200	1,452,400	$9,200	1,476,200	$9,100	3,200,800	$4,200
	**Midwives + Obstetricians**	318,200	$30,700	1,773,800	$5,500	2,517,100	$3,900	4,609,200	$2,100
